# Biosynthesis and characterization of cobalt nanoparticles using combination of different plants and their antimicrobial activity

**DOI:** 10.1042/BSR20230151

**Published:** 2023-07-07

**Authors:** Huma Ali, Yashwant Kumar Yadav, Daoud Ali, Gokhlesh Kumar, Saud Alarifi

**Affiliations:** 1 Department of Chemistry, Maulana Azad National Institute of Technology, Bhopal 462003, India; 2 Department of Mechanical Engineering, Maulana Azad National Institute of Technology, Bhopal 462003, India; 3 Department of Zoology, College of Science, King Saud University, PO Box 2455 Riyadh, 11451, Saudi Arabia; 4 Clinical Division of Fish Medicine, University of Veterinary Medicine Vienna, 1210 Vienna, Austria

**Keywords:** Allium cepa, Allium sativum, antimicrobial activity, cobalt nanoparticles

## Abstract

It has become crucial to biosynthesize efficient, secure, and affordable nanoparticles that we use for the treatment of various infections, including surgical site infection and wound infection, due to the rapid development of microbial resistance to numerous antibiotic drugs. The objective of the present study is to biosynthesize cobalt nanoparticles using an extract from the combined peels of garlic (Allium sativum) and onion (Allium cepa). Scanning electron microscopy (SEM), Fourier-transform infrared spectroscopy (FTIR), and X-ray diffraction were used to confirm the synthesis of cobalt nanoparticle (XRD). Well diffusion was used to measure antimicrobial activity. *Escherichia coli, Proteus, Staphylococcus aureus, Staphylococcus cohnii*, and *Klebsiella pneumonia* were the bacterial strains employed Both the crude prepared extract and the biosynthesized cobalt nanoparticles demonstrated efficacy against all strains of bacteria, but the crude prepared extract displayed a low zone of inhibition ranging from 10 to 13 mm, while the biosynthesized cobalt nanoparticles displayed a high zone of inhibition ranging from 20 to 24 mm.

## Introduction

Recently, biological processes are receiving more focus than physical and chemical methods for the synthesis of nanoparticles [1]. Conventionally, nanoparticles were produced using various physical methods, but these methods had some drawbacks, including high costs, slow production rates, and high energy requirements [2,3]. The production of those materials primarily employs chemical techniques like the sol–gel method and chemical reduction. However, these methods involve the use of cytotoxic chemicals and also result in the creation of hazardous byproducts. As a result, microbes and plant extracts are now used to create nanomaterials [4]. In comparison with microorganisms, the synthesis of nanoparticles using plant extracts is beneficial because it does away with the difficult process of protective cell cultures and can be successfully scaled up for the synthesis of nanoparticles at a large scale. Thus, the development of such methods that are less hazardous, eco-friendly, cost-effective, and clean for the synthesis of nanoparticles continues to be pursued by the scientific community [5]. As a result, current research in the pertinent scientific fields has focused on the synthesis of metal oxide nanoparticles using entirely different plant extracts. Additionally, due to its simple experimental origin and ease of obtaining nanoparticles with different sizes and morphologies, the biogenesis of metal oxide nanoparticles is receiving drastically growing attention [6]. Flavonoids, for example, are believed to play a role in the antioxidant or reducing activities of medicinal plants. The synthetic resin compounds function as singlet oxygen quenchers, reducing agents, hydrogen donators, and metal chelating agents as a result of their oxidation–reduction activities [7,8]. Individual metal nanoparticles are created when phenolic compounds in the plant extract decrease the metal ions [9].

Cobalt is one of the most promising materials among bimetal nanoparticles, and it has long piqued the attention of scientists working in a wide range of disciplines [10,11]. Cobalt nanoparticles in general have amazing magnetic, electrical, and catalytic characteristics that are useful in a variety of scientific and technological applications, including recording media, magnetic sensors, magnetic memories, magnetic fluids, magnetic composites, catalysis, etc. [12–14]. Cobalt nanoparticles are suitable for a variety of static magnet uses due to their high anisotropy fields and saturation magnetization [15]. Additionally, it has recently been discovered that cobalt nanoparticles are useful for radiation absorption uses like the creation of wireless communications and high-frequency circuitry [16,17]. The cobalt nanoparticles also have an inherent benefit in biomedically related areas, such as drug delivery and magnetic resonance imaging [18], where these applications require the highest level of quality and purity to prevent any change in their magnetization or response stability.

The most widely grown vegetable is the onion, also known as the bulb onion or common onion. A type of bulbous angiosperm, garlic is [19]. It was well-known to the ancient Egyptians and has been used as a common medication as well as a culinary flavoring. Each variety of onion and garlic belongs to the Amaryllidaceae family of Allium plants. Onion and garlic’s outer shells are rich sources of antioxidants, vitamins A, C, and E [20]. Onion peels are also rich in flavonoids, especially quercetin, a powerful antioxidant, and anti-inflammatory. Antioxidants found in garlic help the body's defenses against oxidative damage [21]. Garlic supplements at large doses have been shown to increase antioxidant enzymes in people and significantly lessen aerobic stress in people with high blood pressure. There are a lot of minerals in onions. The heart’s wellness may benefit. Rich in antioxidants, also contain anti-cancer chemicals, helps regulate glucose, strengthens the gastrointestinal system, and has a variety of other pharmacologic effects [22].

The use of engineering science to control the growth of microorganisms has grown, along with the number of multidrug-resistant ones, and antimicrobial resistance may be a significant public pathological condition [23]. Due to their effective antimicrobial properties, different nanoparticles in common metals like silver, gold, and iron are the subject of extensive study in this context. An antioxidant is another component that sticks out. Although few studies have examined its use as a bimetal nanoparticle, it has high bioactivity and medication action [24]. Microorganisms that are immune to antibiotics are a major worry and have spread globally. The difficulty and rising cost of care, as well as an increase in the mortality and morbidity rates of those impacted, are some effects of this problem that hurt the economy and the healthcare industry [25]. The creation of compounds and a variety of therapies, awareness campaigns for the prudent use of antibiotics, medical specialty studies of antimicrobial resistance, and monitoring of multidrug-resistant microorganism (MDR) cases are some of the actions associated with the solution. Making novel therapies with natural origins and nanotechnology-related substances, such as biological nanoparticles of silver, gold, and cobalt with antimicrobial properties, is thus a great way to fight this issue.

Given the advantages of using green synthesis to produce nanoparticles and the unquestionable antimicrobial activity of cobalt nanoparticles against various organisms, cobalt nanoparticles are a viable option for developing novel antimicrobials. The goal of this research was to synthesize inexperienced cobalt nanoparticles using a plant extract that was a combination of two different plants and to assess their antimicrobial activity.

## Materials and methods

### Preparation of extracts

Bulbs of onion and garlic were purchased from Piplani market, Bhopal, Madhya Pradesh, India.

Onion and garlic peel were washed with water several times to remove dust and dirt on it and then dried under shade at room temperature and crushed in a grinder. Further, both crushed materials were weighed equally (nearly 30 grams each) and mixed in 1:1 ratio properly, then the aqueous extract was prepared by using meseration process. The extract was filtered by using a filter paper and then store in a refrigerator.

### Biosynthesis of cobalt nanoparticles

As a precursor, cobalt nitrate was used to create nanoparticles. This sodium was dissolved in distilled water to a concentration of 1 millimolar. For the cobalt nanoparticle synthesis, a glass flask containing 30 ml of the previously produced extract, and 70 ml of cobalt salt solution was heated at 80°C for 2.5 h. After that, the solution was chilled to room temperature. The resulting solution was centrifuged for 45 min at 5000 rpm, the supernatant solution was decanted, and the remainder was dried in an incubator at 50°C.

### Characterization of nanoparticles

#### Scanning of electron microscopy (SEM)

Utilizing SEM, morphology and size testing were carried out. About 10 µl aliquots of samples of the synthesized cobalt nanoparticles were placed on slides covered with polylysine (1%) and left to dehydrate after being diluted in deionized water at a concentration of 1 mM. Thus, they were postfixed with 1% tetroxide of osmium (High Grade) for 2 h after being fixed with 0.1 M sodium cacodylate buffer solution (High Grade) (pH 7.2), 2% glutaraldehyde (Sigma-Aldrich), and 2% paraformaldehyde (Merck). The plates were then exposed to progressively higher alcohol concentrations to simulate dehydration. After that, they underwent critical point CO_2_ dehydration, were coated in gold, and underwent SEM analysis. Dynamic light scattering (DLS) analysis of cobalt nanoparticles at a concentration of 100 g/ml revealed their average hydrodynamic size and zeta potential in double-distilled water.

#### Fourier-transform infrared spectroscopy (FTIR)

Synthesized cobalt nanoparticles were analyzed using a Fourier-transform infrared spectrophotometer in the range of 600 to 4,000 cm^−1^ with a resolution of 4 cm^−1^ and 64 scans in transmittance mode.

#### X-ray diffraction (XRD)

This analysis was performed on a diffractometer at room temperature, 40 kV, 20 mA, and using CuKα radiation. A Mythen 1 K detector was employed to collect X-ray photons from a powder sample at a time interval of 2 h.

### Test microorganisms

*Escherichia coli* (MTCC No. 1698), Proteus (MTCC No. 658), *Staphylococcus aureus* (MTCC No. 9886), *Staphylococcus cohnii* (MTCC No. 10219), and *Klebsiella pneumoniae* (MTCC No. 3040) were chosed based on their clinical and pharmacological importance. All the strains were purchased from Institute of Microbial Technology, Chandigarh, and used for evaluating antimicrobial activity.

### Growth media preparation

Nutrient Agar for bacterial strains was prepared according to the following standard procedure.

#### Purpose

Nutrient Agar is used for the cultivation of a wide variety of non-fastidious bacteria. It was originally developed in recognition of the need for a standardized medium for use in the examination of water and wastewater, dairy products, and various foods.

Procedure for preparation: Suspend 6 g of the powder in 250 ml of purified water. Heat with frequent agitation and boil for 1 min to completely dissolve the powder. Autoclave at 121°C for 15 min and cool to 45–50°C. Pour 15–20 ml of the ready media into sterile 20 ml glass universal tubes. Leave to stand for 30 min to solidify, resting the tubes leaning at 30–60°C to produce the slope effects in the tubes. Sterility of the media was checked by incubating at 37°C for 24 h and was stored at 40 oc for further use but not more than 2 weeks. The composition of Nutrient Agar media includes Peptone (5 g/L), Beef extract (1.50 g/L), Yeast extract (1.50 g/L), sodium chloride (5.0 g/L), and distilled water (1000 ml).

### Antimicrobial assay

Nutrient Agar media was sterilized at 121°C and 15 lbs pressure for 15 min then media was poured in to petri dishes. The solidified plates were bored with 5 mm diameter cork bearer. The plates with well were used for antimicrobial assay. The crude extract and synthesized cobalt nanoparticles of concentration 200, 150, 100, and 50 µg/ml was tested *Escherichia coli, Proteus, Staphylococcus aureus, Staphylococcus cohnii,* and *Klebsiella pneumonia* for antimicrobial activity by using well diffusion method [26,27].

The prepared culture plates were inoculated with different selected strains of bacteria using the streak plate method. Different samples were poured into the well using sterile syringe. The plates were incubated at 37°C for 24 h for bacterial activity. Each concentration of the crude extract and synthesized cobalt nanoparticles was tested against different bacterial strains. The zone of inhibition was calculated by measuring the diameter of the inhibition zone around the well including the well diameter. The readings were calculated in four different fixed directions and average values were calculated.

### Statistical analysis

Data were explored using the software Primer. Analysis of variance was carried out between the different concentrations, with *P*<0.05 statistically significant. The results were obtained as mean and standard deviation (SD).

## Results

**Figure 1:**
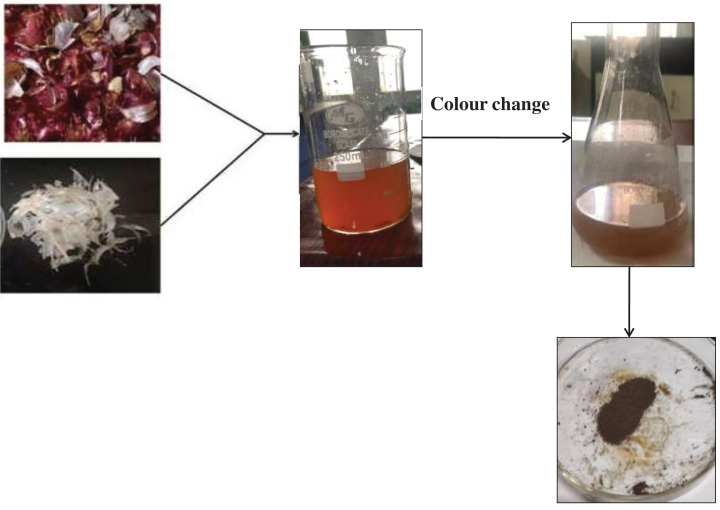
Biosynthesis of cobalt nanoparticles from the combine extract of onion and garlic peel

The solution color changed from orange to dark brown, Figure 1 shows that cobalt nanoparticles have formed. For 4 days, the biogenesis of cobalt nanoparticles was monitored by looking at the color of the solution. The diminution is indicated by the orange’s transformation into grey. The third day’s color change intensity was quite strong, and no change was seen between the third and fourth days, indicating that the biosynthesis of cobalt nanoparticles had come to an end. Dark color solutions typically have spherical, circular, or rod-shaped nanoparticles, which can be determined by the obtained color.

**Figure 2:**
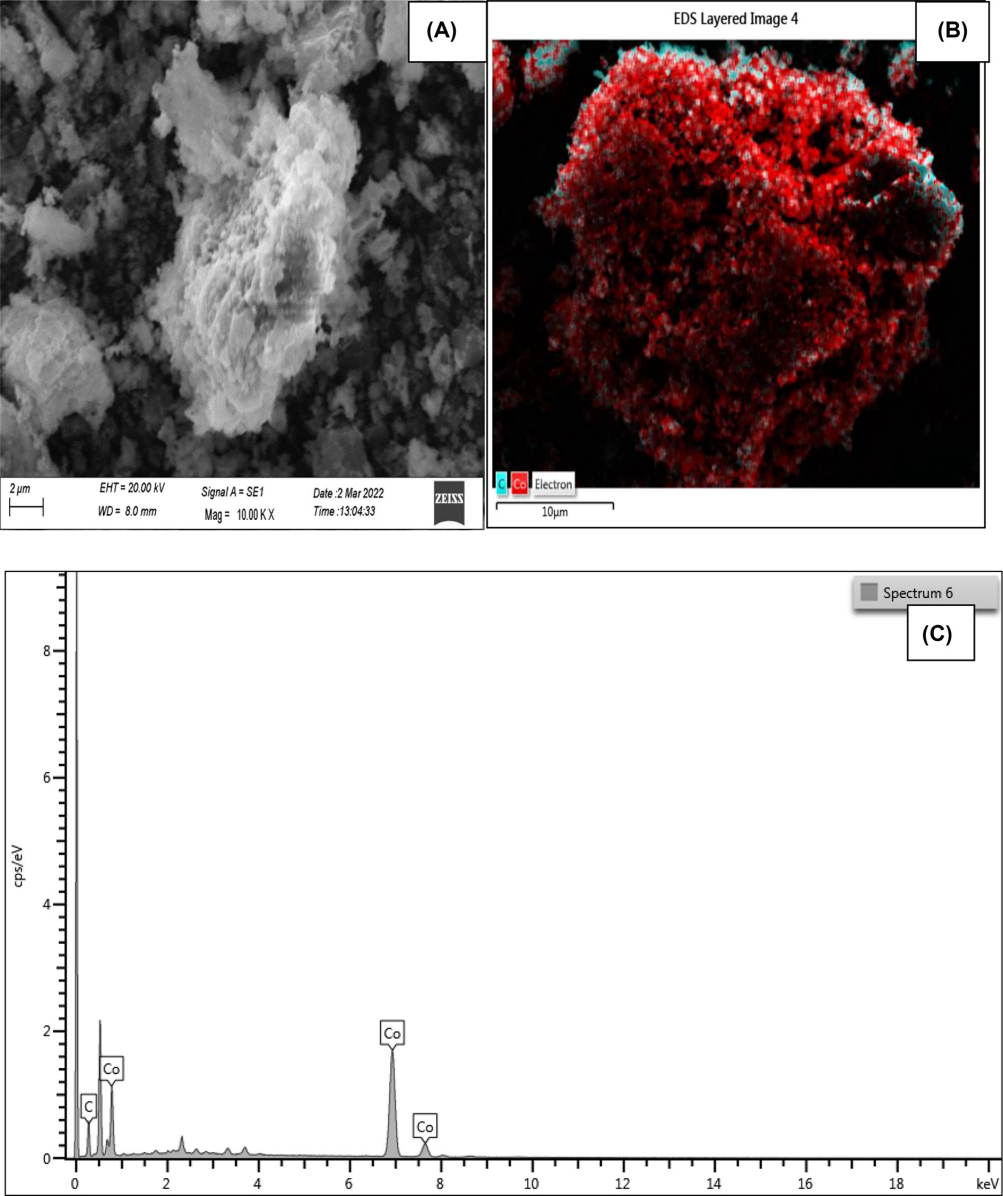
Analysis of biosynthesized cobalt nanoparticles (**A**) SEM image, (**B**) EDS-layered image, and (**C**) EDS graph.

**Figure 3:**
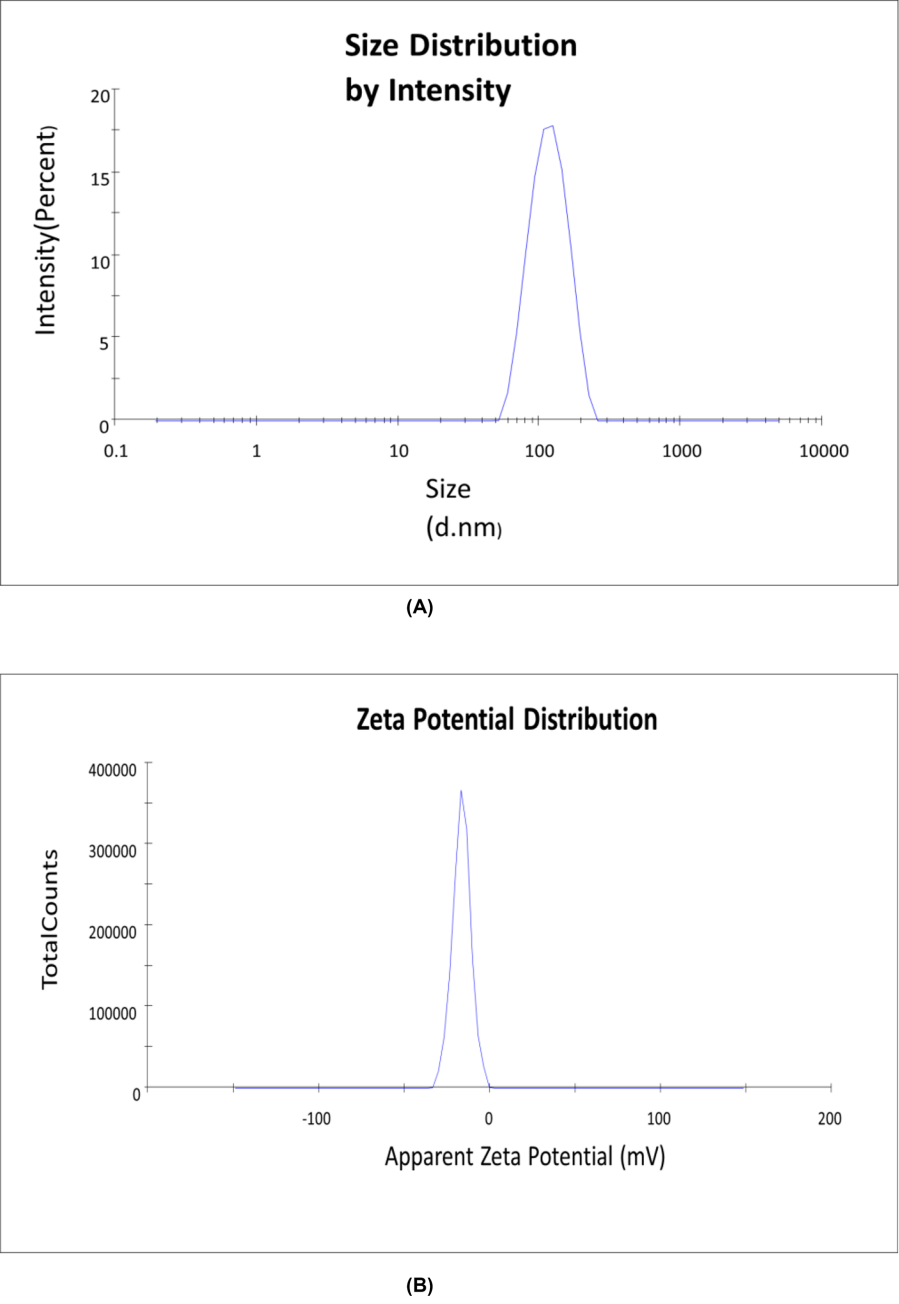
Size analysis of biosynthesized cobalt nanoparticles (**A**) DLS measurements of particle size distribution and (**B**) charge zeta potential.

The SEM picture of cobalt nanoparticles is shown in Figure 2A, which clearly demonstrates that they have a nanoparticle-like morphology that specifies well-uniform particles with a narrow size distribution that lies in the 20–30 nm range. The smooth surface of biosynthesized nanoparticles makes it easier for them to make greater contact with bacterial cell walls, increasing their capacity to kill germs. Smooth-surfaced nanoparticle behavior in this manner has already been proven. EDS analysis was used to evaluate the cobalt nanoparticles’ elemental makeup. Figure 2B demonstrated that the presence of cobalt is shown by the red color, while the presence of carbon is indicated by the blue color, which is caused by some ash and impurities. The primary peaks in Figure 2C represent the Co of the synthesized NPs. According to Table 1, the nanoparticles’ elemental composition includes 27.06% carbon and 72.94% cobalt. The EDX analysis’s compositional results and theoretically predicted values are in good agreement, demonstrating the nanoparticles’ strong compositional homogeneity. The suspension of cobalt nanoparticles was prepared in Mille Q water and their size was determined using a zeta sizer, size and zeta potential were found to be 150 ± 1.0 nm and −20.4 ± 3.8 mV, Figure 3.

**Table 1:** Facts of elemental analysis of biosynthesized cobalt nanoparticles

Spectrum label	Spectrum 6
C	27.06
Co	72.94
Total	100.00

**Figure 4:**
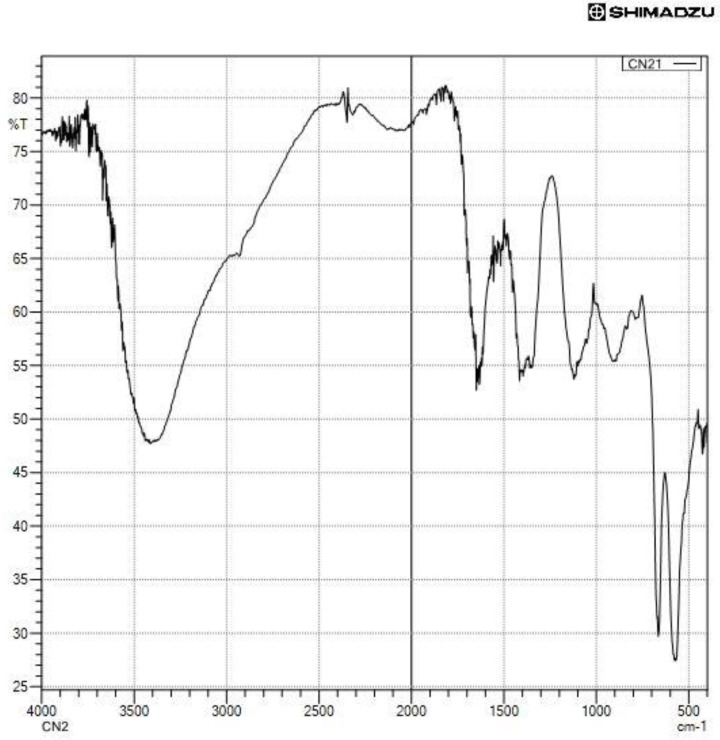
FTIR-spectra of biosynthesized nanoparticles

The numerous functional groups that are present in the biosynthesized cobalt nanoparticles and serve as capping and stabilising agents were identified using FT-IR analysis. Figure 4 displays the IR spectra of biosynthesized nanoparticles. The typical peak of the hydroxyl group of phenolic compounds was measured approximately 3400 cm^−1^. Other significant peaks at 1622, 1300, 1100, and 400 cm^−1^, respectively, can be attributed to the carbonyl group, amide group, C–O of alcohols or phenols, and Co_3_O_4_.

**Figure 5:**
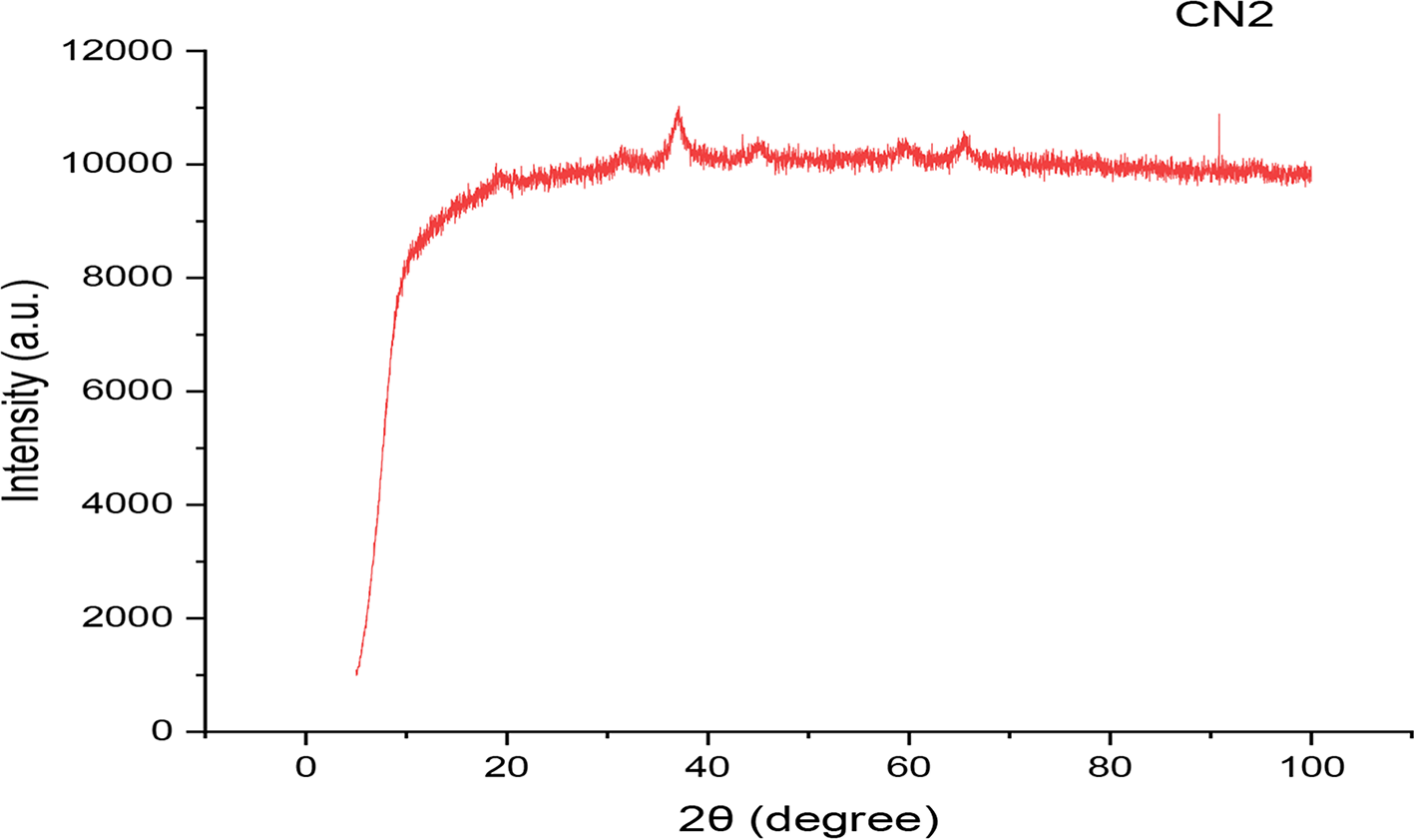
XRD spectra of biosynthesized cobalt nanoparticles

By using powder x-ray diffraction, the effective biosynthesis of cobalt nanoparticles was verified (XRD). Figure 5, shows that the resultant diffraction pattern lacks strong peaks, indicating that the produced nanoparticles are not sufficiently crystalline. There are, however, a few sporadic faint peaks at 2 values of 30.3 and 90, or (210) and (311), respectively. The particle size was discovered to be between 20 and 30 nm.

**Figure 6:**
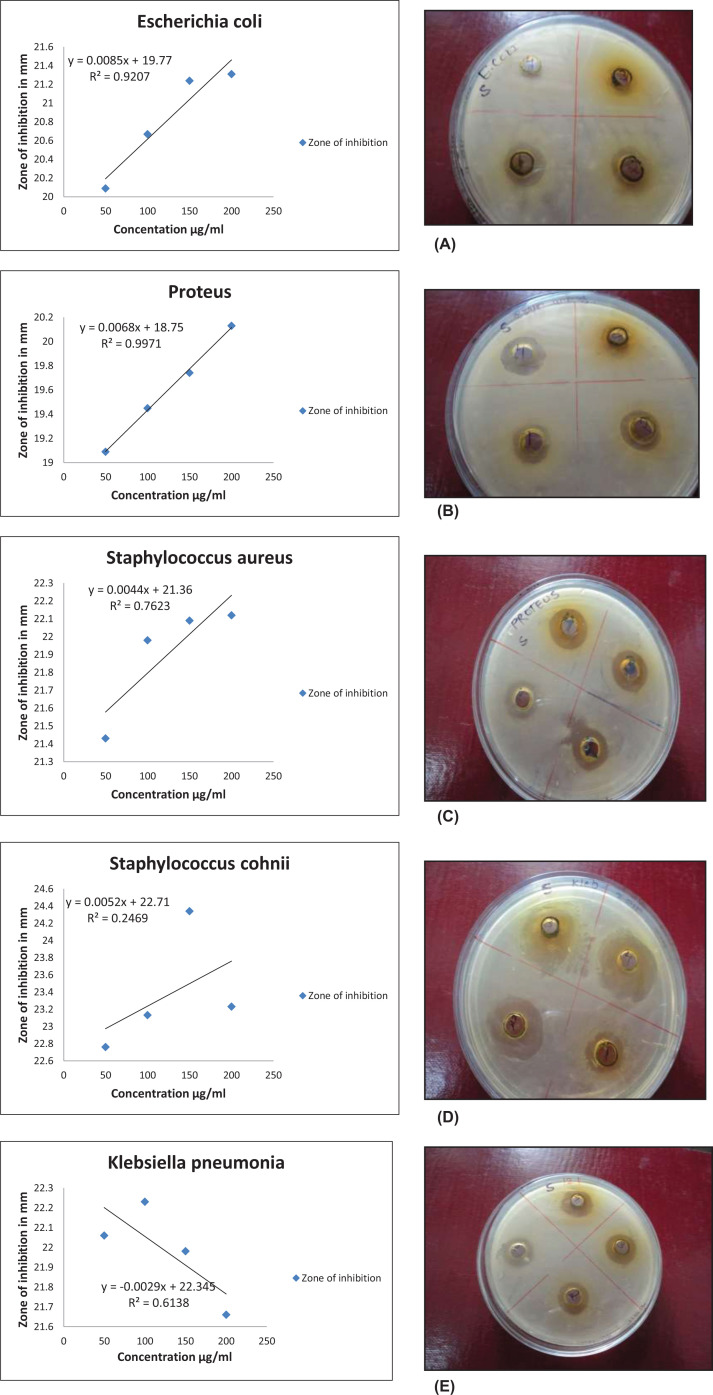
Line regression curve for the determination of IC50 and Zone of inhibition of biosynthesized cobalt nanoparticles (**A**) *Escherichia coli,* (**B**) *Proteus,* (**C**) *Staphylococcus aureus,* (**D**) *Staphylococcus cohnii,* and (**E**) *Klebsiella pneumonia*.

Different bacterial strains were used to test the antibacterial properties of crude extract, prepared crude extract, and cobalt nanoparticles. The zone of inhibition for *Escherichia coli, Proteus, Staphylococcus aureus, Staphylococcus cohnii*, and *Klebsiella pneumonia* is shown in Tables 2 and 3 for the crude extract and cobalt nanoparticles, Figure 6. Line regression curve for the determination of IC50 of biosynthesized cobalt nanoparticles is shown in Figure 6, and it was found be 3.55, 4.59, 6.50, 5.24, and 9.53 mg/ml for *Escherichia coli, Proteus, Staphylococcus aureus, Staphylococcus cohnii*, and *Klebsiella pneumonia,* respectively. Numerous diseases can be brought on by microbial infections in living things. Against all of the aforementioned bacteria, the bio produced cobalt nanoparticles showed a higher zone of inhibition than the crude extract did at a similar dose. An increase in surface oxide ion concentration with increasing nanoparticle surface area led to more efficient bacterial cell wall and cytoplasm membrane breakdown.

**Table 2:** Showing zone of inhibition in mm of crude extract of onion and garlic (seperately) against different microorganism

Name of bacterial strains	Zone of inhibition of crude onion extract (in mm)	Zone of inhibition of crude garlic extract (in mm)
	200 µg/ml	200 µg/ml
*Escherichia coli*	9.08 ± 0.19	10.21 ± 0.12
*Proteus*	10.34 ± 0.23	9.43 ± 0.32
*Staphylococcus aureus*	10.78 ± 0.12	10.14 ± 0.45
*Staphylococcus cohnii*	11.69 ± 0.35	11.05 ± 0.38
*Klebsiella pneumonia*	11.54 ± 0.54	12.19 ± 0.15

**Table 3:** Showing zone of inhibition in mm of crude prepared extract and biosynthesized cobalt nanoparticles against different microorganism

Name of bacterial strains	Zone of inhibition of crude prepared extract (in mm)				Zone of inhibition of synthesized cobalt nanoparticles (in mm)			
	200 µg/ml	150 µg/ml	100 µg/ml	50 µg/ml	200 µg/ml	150 µg/ml	100 µg/ml	50 µg/ml
*Escherichia coli*	10.11 ± 0.12^*^	10.08 ± 0.81	9.86 ± 0.21	9.54 ± 0.34	21.31 ± 0.02	21.24 ± 0.19	20.67 ± 0.05	20.09 ± 0.65
*Proteus*	12.34 ± 0.28^*^	11.38 ± 0.56	11.24 ± 0.04	10.6 ± 0.18	20.13 ± 0.65	19.74 ± 0.23	19.45 ± 0.78	19.09 ± 0.13
*Staphylococcus aureus*	11.67 ± 0.89^*^	11.23 ± 0.67	10.98 ± 0.54	10.7 ± 0.67	22.12 ± 0.34	22.09 ± 0.12	21.98 ± 0.56	21.43 ± 0.23
*Staphylococcus cohnii*	13.45 ± 0.56^*^	12.76 ± 0.87	12.34 ± 0.82	11.6 ± 0.78	23.23 ± 0.24	24.34 ± 0.65	23.13 ± 0.18	22.76 ± 0.45
*Klebsiella pneumonia*	12.76 ± 1.29^*^	13.45 ± 0.69	12.19 ± 0.13	12.74 ± 0.40	21.66 ± 0.45	21.98 ± 0.76	22.23 ± 0.30	22.06 ± 0.15

The results were obtained as mean and standard deviation (SD).

* *P*<0.05 is taken as statistically significant.

## Discussion

As per our data up to now, the current study is that the initial to synthesize cobalt nanoparticles employing a combination of two plant extracts, one of that is onion and another one is garlic. Combination of more than one plant gives better results as compared with the individual one due to the synergistic effect. Several studies proved this fact [28]. Cobalt nanoparticles are generally made by chemical synthesis. Many chemical ways were applied for cobalt nanoparticles synthesis. The primary standard methods for chemical synthesis are the chemical reduction of oxides [29], evaporation–condensation methods [30,31], sol–gel, and plasma–chemical synthesis. Chemical modes are widely used for cobalt nanoparticle production despite their multistep character [32]. These ways are characterised by technological simplicity, and additionally by the flexibility to regulate the synthesis method at every stage by varied synthesis conditions like temperature, pH, concentrations of reagents, etc [33]. The parameters of the synthesis are varied for getting the greatest nanoparticles with needed properties like part composition, dispersity, and morphology. Cobalt nanoparticles are obtained by the chemical reduction of salts in alkali solutions, followed by chemical element reduction. However, the influence of the synthesis conditions on the properties of the obtained nanoparticles, like particle size distribution and part composition, remains unclear. Synthesis of cobalt nanoparticles from mixed plant extracts is a smaller amount cytotoxic, not generating cytotoxic compounds to the setting.

The plants utilized in this study (onion and garlic) are accessible, easily found in markets and houses throughout Bharat, and have low prices compared with the chemical synthesis product. Onion and garlic have high amounts of polyphenolic compounds like flavonoid that has antimicrobial activity against some completely different microbic strains. The suspension was monodispersed and produced strong colloidal stability, according to the DLS analysis, which also showed a unimodal size distribution with polydispersity indices. This finding indicates that the biosynthesized nanoparticles surface picked up a negative charge and extensively distributed throughout the liquid. The produced nanoparticles in the suspensions were successfully stabilised as a result of the indicated negative value. Additionally, because the average size is a gauge of hydrodynamic size, its value reveals the presence of nanoparticles as well as any solvent molecules associated with the tumbling particle [34].

The bacteria tested during this study have nice medical significance. *S. aureus* is often found on skin microbiota; but, skin lesions will promote bacterial entry to the blood, inflicting carditis, pneumonia, osteitis, and different infections [35,36]. This infectious agent is found in hospitals and different communities. cocci cohnii may be a coagulase-negative member of the bacterial genus Staphylococcus consisting of clustered cocci.

The species typically lives on human skin; clinical isolates have shown high levels of antibiotic resistance. *Proteus* caryophylloid dicot genus may be a facultative being with swarming motility and a capability to self-elongate and secrete a sugar that permits it to connect to and move on surfaces like catheters, endovenous lines, and different medical instrumentation. Ninety percent of *Proteus* infections occur as a result of *Proteus mirabilis*, and these are thought of as community-acquired infections. Although not a typical reason for healthcare facility infections, Proteus species have additionally been shown to cause infection from the inhabited skin and oral membrane of patients and personnel operating in an exceedingly hospital or long-run care facility. Urinary tract infections (UTIs) occur as a result of bacterial migration on the membrane sheath of the tube or up the tube lumen from contaminated piss. Different factors that will increase the chance of infection by the P. caryophylloid dicot genus embrace feminine sex, a longer length of catheterization, improper tube improvement or care, underlying unhealthiness, and lack of accessibility of general antibiotics.

*E. coli* is usually found within the enteral microbiota; but, unhealthful strains are the foremost important reason of diseases in humans and also the biggest chargeable for tract infections. What is more, *E. coli* immune to many cephalosporins and fluoroquinolones, antibiotics commonly utilized in human medication, have already been according.

Klebsiella pneumonia is quickly developing multidrug resistant (MDR) strains and generally creates a significant threat to the patients attributable to an increased morbidity thanks to the reduced effectiveness of medical care choices. *K. pneumoniae* is understood to be chargeable for community noninheritable infections though recently it’s habitually discovered as a serious reason for hospital noninheritable pathogens. *K. pneumoniae* has been discovered to develop resistance to antibiotics a lot of simply than most bacteria through the production of enzymes like extended spectrum β-lactamase (ESBL) and carbapenemase [37–39].

Cobalt nanoparticles synthesized within the gift study incontestable antimicrobial activity against higher than-represented strains, namely, *S. aureus*, cocci cohnii, Proteus caryophylloid dicot genus, *E. coli* and enteric bacteria respiratory disorder. Synthesis of iron and atomic number 27 nanoparticles of garlic extract and their other application were already according to different research workers. During this study, an inexperienced procedure is applied for the preparation of atomic number 27 nanoparticles using a mixed extract of Onion and Garlic peel that is tested against various bacterial strains and also the results showed that the biosynthesized atomic number 27 nanoparticles possessed smart bacterial action against all organisms because of the synergistic effect as the cobalt nanoparticles was prepared by using two different natural material i.e. onion and garlic peel.

## Conclusion

The effective biosynthesis of cobalt nanoparticles was demonstrated by SEM, EDS, FTIR, and XDR. Depending on the studied bacteria and the different exposure concentrations, cobalt nanoparticles showed evidence of bacterial activity. Biosynthesized cobalt nanoparticles had significant antimicrobial action against all strains of bacteria, while this activity was less potent than crudely produced extract. Given that bacterial resistance is a growing problem for public health, substances like the cobalt nanoparticles that were biosynthesized in this study may replace antibacterial control. Therefore, cobalt nanoparticles can be put to lotions, ointments, and gels to treat burns and wounds due to their antibacterial function. They can be connected to probiotics and utilized as growth enhancers in poultry. Cobalt nanoparticles can also be utilized as a soil additive and in the creation of particular antibacterial products for various microorganisms. The utilization of the nanoparticles created in this work has several potential applications.

## Data Availability

The materials and raw data are available from the authors upon request.

## Abbreviations

ESBL: extended spectrum β-lactamase
FTIR: Fourier-transform infrared spectroscopy
MDR: multidrug resistant
SEM: scanning electron microscopy
UTI: urinary tract infection
XRD: X-ray diffraction
